# Physiologically‐Based Pharmacokinetic Modeling to Investigate Piperaquine Exposure in Pregnant Women Using an Individualized Profile Approach

**DOI:** 10.1111/cts.70589

**Published:** 2026-05-12

**Authors:** Sonia Khier, Myriam El Gaaloul, Joel Tarning, Nada Abla

**Affiliations:** ^1^ Institut des Biomolécules Max Mousseron (IBMM), CNRS, UMR5247 Montpellier University Montpellier France; ^2^ Pharmacokinetics and Pharmacometrics Department, Faculty of Pharmaceutical and Biological Sciences Montpellier University Montpellier France; ^3^ MMV Medicines for Malaria Venture Geneva Switzerland; ^4^ Mahidol Oxford Tropical Medicine Research Unit, Faculty of Tropical Medicine Mahidol University Bangkok Thailand; ^5^ Centre for Tropical Medicine and Global Health, Nuffield Department of Medicine University of Oxford Oxford UK

## Abstract

Malaria in pregnancy poses significant risks to the mother, fetus, and neonate, necessitating effective treatment and prevention strategies. This study explored the application of an individualized (‘virtual twin’) physiologically‐based pharmacokinetic (PBPK) modeling approach to the antimalarial drug piperaquine in pregnant women. The piperaquine PBPK model was previously developed and validated within Simcyp for a healthy non‐pregnant population. Individualized profiles considering patient age, gestational age, and piperaquine dose were generated using the Simcyp pregnancy population for 36 patients from two clinical trials, including 12 Sudanese and 24 Thai pregnant women with malaria. Model predictive performance was assessed by comparing simulated piperaquine concentrations with observed clinical data. Mean body weight was higher in the virtual vs. the observed population, requiring adjustment of exposures to reflect the mg/kg dose in malaria patients. Results showed good model performance for exposures up to Day 3, with AUC predicted:observed ratios within 0.5 to 2.0 for all Sudanese women in the second (5/5) or third trimester (7/7) and for 14/16 Thai women in their second and 8/8 in their third trimester. The model overestimated clearance in the Sudanese population by ~1.7‐fold, leading to underestimation of Day 7 plasma concentrations. This methodological investigation demonstrated the potential of an individualized PBPK approach to inform antimalarial drug pharmacokinetics in pregnancy. Further model refinement is needed to support dose adjustment, including verification of additional individualization factors against clinical data sets. This highlights the need for generating and sharing pharmacokinetic and disease‐specific demographic data from pregnant women in malaria‐endemic countries.

## Study Highlights

- What is the current knowledge on the topic?
  ○ Malaria in pregnancy is a significant public health challenge, with the mother, fetus, and neonate at risk of adverse outcomes. Exclusion from clinical trials for fear of causing harm prevents access to medicines at appropriate doses for this population.
- What question did this study address?
  ○ Piperaquine is one of two compounds included in the widely used antimalarial combination therapy, dihydroartemisinin‐piperaquine phosphate. This study evaluated an individualized profile approach for a physiologically‐based pharmacokinetic (PBPK) model of piperaquine in a healthy Caucasian pregnant population using observed data from Sudanese and Thai pregnant women with malaria.
- What does this study add to our knowledge?
  ○ This study, using piperaquine as an example, is the first to apply an individualized (‘virtual twin’) PBPK approach to antimalarial pharmacokinetics in pregnancy. It provides a practical workflow for developing and evaluating subject‐specific models and highlights the strengths and limitations of the approach.
- How might this change drug discovery, development, and/or therapeutics?
  ○ This approach accounts for individual variability in drug pharmacokinetics during pregnancy. Fully validated models would support dose optimization in pregnant women and inform the design of clinical trials for antimalarial drugs in this underserved and high‐risk population.

## 1 Introduction

Malaria in pregnancy is a significant public health challenge in endemic regions, particularly sub‐Saharan Africa, where an estimated 13 million pregnancies were exposed to malaria infection in 2024 [1]. Pregnant women are susceptible to severe disease because of pregnancy‐induced changes to the immune system [2]. Infections are not always detectable using conventional diagnostic techniques, as parasites are sequestered in the placenta, causing impaired fetal nutrition and retarding growth [3]. The infection is associated with an increased risk of maternal anemia, low birth weight, preterm delivery, and maternal and neonatal death [4].

Malaria prevention during pregnancy includes vector control and chemoprevention through intermittent preventive treatment with antimalarial drugs [5]. Sulfadoxine‐pyrimethamine has been widely used for chemoprevention in pregnancy and has been shown to improve outcomes [5]. However, it cannot be used before the thirteenth week of pregnancy [5], and its effectiveness is threatened by the spread of drug resistance in Africa [1].

Well‐tolerated and effective antimalarial drugs are also needed for case management of malaria in pregnant women. Artemisinin‐based combination therapy is the first‐line treatment for malaria, but only artemether‐lumefantrine is recommended by the WHO for use from the first trimester in pregnancy [5]. This recommendation was made more than twenty years after artemether‐lumefantrine was introduced as a first‐line malaria drug for use in adults. Despite the clear medical need, pregnant women continue to be a neglected population in the provision of antimalarial treatment and chemoprevention [6].

Pregnant women are almost always excluded from clinical trials [6], and there is no regulatory mandate requiring the inclusion of this population in drug development studies. This approach is rooted in the precautionary principle aimed at shielding both the mother and fetus from potential research‐related harm. As a result, critical data, including pharmacokinetics (PK), which support dose optimization and risk–benefit assessments, remain largely unavailable for pregnant populations. In most cases, information about medication used during pregnancy is derived retrospectively through observational data from registries tracking off‐label exposure. This reliance on *post hoc* data collection delays the availability of evidence necessary to guide clinical decision‐making, often leaving healthcare providers without the tools to make informed therapeutic choices. To mitigate these deficiencies, innovative strategies are needed that incorporate risk abatement while generating timely, high‐quality evidence to support the health of both mother and fetus [6].

Maternal physiological changes occur during the perinatal life stages. Some of these changes have been well described and incorporated into physiologically‐based PK (PBPK) models [7, 8]. PBPK modeling may be useful to predict PK exposure in the context of pregnancy, combining the effects of multiple physiological parameters on drug PK and considering multiple compartments corresponding to the different organs or tissues in the body. These compartments are connected by flow rates that reflect the circulating blood system. Within the Simcyp PBPK simulator, a pregnancy model has been developed, accounting for the known physiological changes that occur during pregnancy. However, this model was developed using data from healthy, predominantly Caucasian women, and it may not be generalizable to populations with different ethnic backgrounds or health conditions [9], specifically women with malaria who may have altered physiology [10, 11]. Besides, the model was validated using drugs that are not antimalarials [12, 13].

The ‘virtual twin’ approach involves modification of the PBPK model to incorporate individualized data from real‐life patients [14, 15, 16, 17, 18, 19, 20, 21]. This strategy aims to improve the accuracy of the PK predictions by reflecting the physiological characteristics of actual patients, allowing comparison of the simulations with individuals, rather than across a population. However, there is no consensus on the specific characteristics that should be individualized, and different contexts vary in the availability and/or relevance of individualized data, such as demographic characteristics, environmental factors, hepatic or renal function, clinical laboratory parameters, metabolizing enzyme phenotypes, or other genetic traits [14, 15, 16, 17, 18, 19, 20, 21]. Recognizing that no model can fully incorporate all the individual traits that could be relevant, we use the term ‘individualized profile’. Such studies can be applied to dose optimization, linking to drug safety, inter‐individual population variability, and the design of clinical trials. However, data are not available for all relevant pregnancy‐related physiological changes. Also, in the context of pregnancy in malaria, there are very few compounds with sufficient PK data across all trimesters.

Piperaquine is an approved antimalarial drug co‐formulated with dihydroartemisinin (DHA‐PPQ), recommended for the treatment of uncomplicated *Plasmodium falciparum* malaria. Piperaquine has a very long half‐life (> 20 days) [22] and has also been investigated for chemoprevention in children [23] and in pregnant women from the second trimester in pregnancy [24]. Thus, the available PK data sets for piperaquine are rich enough to allow an individualized approach.

In this study, we evaluated the predictive performance of a PBPK model for piperaquine exposures using patient profiles individualized for age, gestational age, and piperaquine dose. These parameters were chosen as the most accessible and simplest approach to assess performance in this methodological investigation. Other characteristics were generated using the Monte Carlo approach using the standard healthy Simcyp pregnancy population. We examined whether the simulated piperaquine concentration at the therapeutic dosage in pregnant women was adequately described relative to pregnant women with malaria from Sudan and Thailand.

## 2 Methods

### 2.1 Observed Data

To identify articles reporting rich PK observations for piperaquine (drug concentration vs. time) in pregnant women, a literature search was conducted on 13th October 2023 using PubMed with the following search terms: [pregnant] AND [pharmacokinetic] AND [piperaquine]. Of the 30 articles returned, two included adequate descriptions of PK for piperaquine following oral dosing in pregnant women with uncomplicated *P. falciparum* malaria and had full PK datasets available [25, 26]. These datasets provided the observed data for the PBPK analysis. The characteristics of both studies are described in Table 1. In the study in Sudanese women, standard dosing of DHA‐PPQ was administered once a day for three days, and blood samples for PK analysis were collected at 0, 1.5, 4, 8, 24, 25.5, 28, 32, 48, 49, 50, 51, 52, 54, 56, 60, and 72 h and then at Days 5, 7, 14, 21, 28, 35, 42, 49, 56, and 63 after starting treatment [25]. In the study in Thai women, standard dosing of DHA‐PPQ was administered once daily for three days, and samples taken at 0, 0.5, 1.5, 4, 8 (before the second dose), 24.5, 25.5, 28, and 32 (before the third dose), and 48.25, 48.5, 49, 50, 51, 52, 54, 56, 60, and 72 h and at Days 5, 7, 14, 21, 28, 35, 42, 49, 56, 63, 77, and 84 after starting treatment [26]. A retrospective analysis was performed using the observed concentrations vs. time data. Area under the time course curve (AUC) and maximum concentration (C_max_) were derived by non‐compartmental analysis with PKanalix (version 2021R1, Lixoft SAS, Antony, France) and considered the reference values for drug exposure.

**TABLE 1:** Demographic and treatment characteristics of pregnant women enrolled in clinical trials used for the observed data set. DHA‐PPQ, dihydroartemisinin‐piperaquine phosphate; NA: Not available.

Characteristic	Adam, et al. (2012) [25]	Rijken, et al. (2011) [26]
Country	Sudan	Thailand
Number of women	12	24
Mean body weight, kg [range]	59.6 [50–72]	49.2 [36–58]
Mean age, years [range]	24.5 [18–33]	28.1 [18–43]
Gestational age		
Second trimester, 13–28 weeks, *n* (%) [range]	5 (41.6) [15.3–26]	16 (67) [13.1–27.4]
Third trimester, 28–40 weeks, *n* (%) [range]	7 (58.3) [26–40.1]	8 (33) [28.5–33.4]
Clinical status	Uncomplicated *P. falciparum* malaria	Uncomplicated *P. falciparum* malaria (*n* = 20) Uncomplicated mixed *P. falciparum* and *P. vivax* malaria (*n* = 4)
Formulation	DHA‐PPQ (DuoCotecxin), Beijing, People's Republic of China	DHA‐PPQ (Holleypharm, People's Republic of China)
Dose regimen	Once a day for three days	Once a day for three days
Mean DHA dose, mg/kg [range]^a^	2.20 [2.00–2.35]	2.24 [2.04–2.44]
Mean PPQ dose, mg/kg [range]^a^	17.57 [16.00–18.82]	17.91 [16.33–19.51]
Mean piperaquine base dose, mg/kg [range]^a^	10.24 [9.33–10.97]	10.44 [9.52–11.38]
Fasted/fed	Fasted	NA

^a^ Actual doses are shown (personal communication JT), not target doses.

### 2.2 PBPK Individualized Model Approach

The piperaquine PBPK model for a healthy, non‐pregnant population dosed in a fasted state was developed and validated within the Simcyp simulator (Certara UK Ltd., Sheffield, UK), as previously reported [27]. The physicochemical and PK input parameters for piperaquine and sources are provided in the Table S1.

**FIGURE 1:**
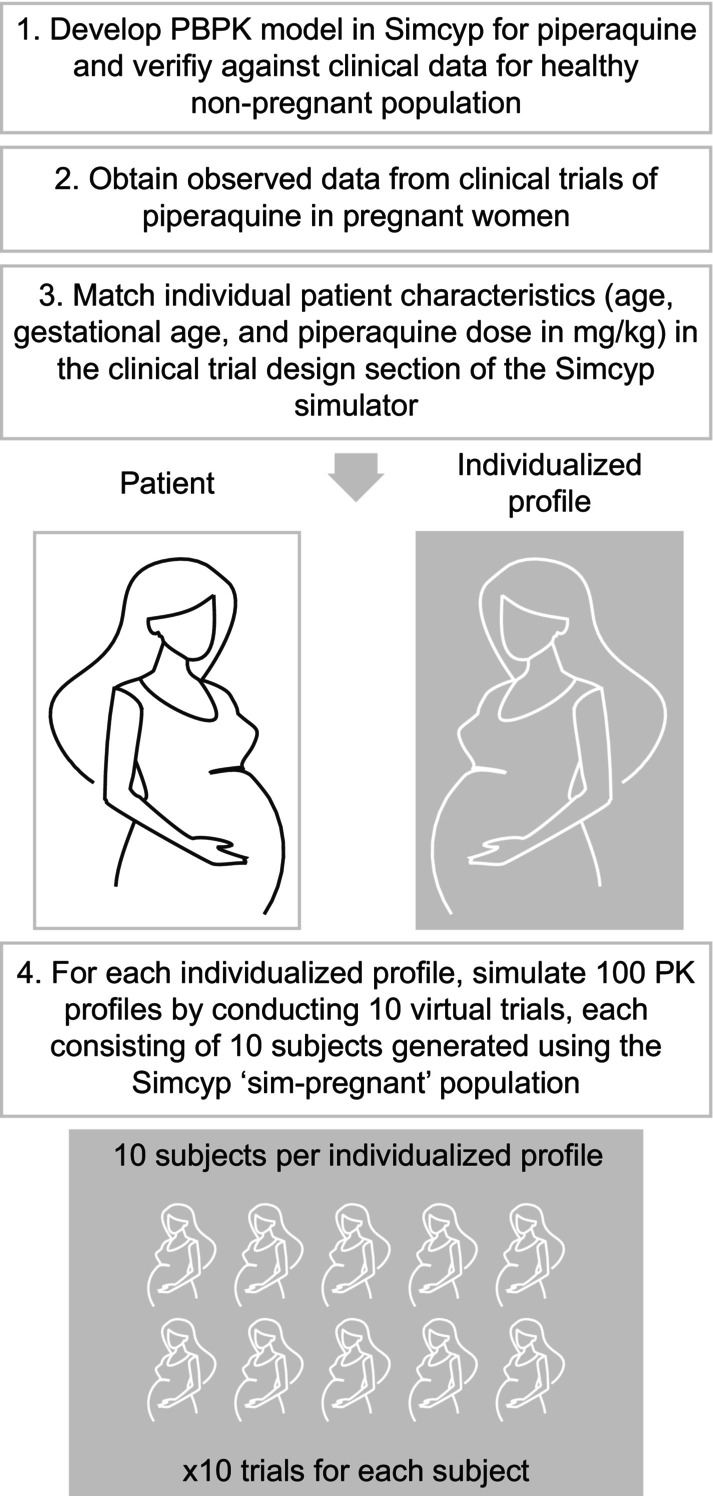
Overview of the workflow for physiologically‐based pharmacokinetic modeling of piperaquine for a pregnant population using an individualized profile (‘virtual twin’) approach.

The simplest approach for constructing an individualized model within the Simcyp platform was adopted using easily accessible data. For each of the 36 women in the observed dataset, an individualized demographic profile was created by matching age, gestational age, and actual piperaquine dose administered (in mg/kg) within the clinical trial design module (Table 1) [25, 26]. Body weight and other physiological characteristics outside the clinical trial design module (e.g., height, body surface area, organ weights [brain, kidney, liver], body mass index, cardiac output, hematocrit, human serum albumin, alpha‐1 acid glycoprotein, serum creatinine, glomerular filtration rate and renal function) were stochastically generated based on the ‘Sim‐Pregnant’ population Simcyp library (version 21) which represents a healthy population of Caucasian pregnant women [28]. For each of the 36 individualized profiles, 10 subjects were generated. For each of these 10 subjects, 10 virtual trials were conducted, resulting in 100 simulated PK profiles per individual patient (Figure 1).

The simulated body weights for each virtual individual were used to convert piperaquine mg/kg doses to absolute mg doses. To account for differences in body weight between the simulated and observed populations, model‐derived AUC values for the first and third doses were normalized using the following equation:

(1) $$\begin{array}{c} {\mathrm{AUC}}_{\text{normalized}}={\mathrm{AUC}}_{1\;\mathrm{mg}}=\frac{{\mathrm{AUC}}_{0-\tau }\;\text{predicted},\text{PBPK model}}{\text{Dose administrated}} \end{array}$$

Assuming linear pharmacokinetics for the doses under investigation, corrected piperaquine exposure ratios (*R*_AUC_) were subsequently calculated to allow direct comparison between simulated and observed AUCs.

### 2.3 Model Assessment

To assess the predictive performance of the PBPK model, for each observed/individualized profile pair, the following exposure ratios were calculated for the first (0–24 h) and the last day (48–72 h) of treatment, as follows:

(2) $$\begin{array}{c} R_{\mathrm{AUC}}=\frac{{\mathrm{AUC}}_{0-\tau }\;\text{predicted},\text{PBPK model}}{{\mathrm{AUC}}_{0-\tau }\;\text{observed},\mathrm{NCA}} \end{array}$$

where ‘AUC_0‐τ_ predicted, PBPK model’ is the mean exposure of the 100 simulated concentration–time profiles from the individualized demographic profile, and ‘AUC_0‐τ_ observed, NCA’ is the exposure values obtained for the patient by non‐compartmental analysis of the observed clinical data. The overall performance of simulations was assessed by the mean fold error (MFE) for AUC and C_max_ for all the simulations and observations as follows:

(3) $$\mathrm{MFE}=\frac{\text{Mean values predicted}}{\text{Mean value observed}}$$

The model was accepted if all predicted exposure values were within two‐fold of the corresponding observed values, i.e., MFE 0.5 to 2.0 [19].

Venous plasma piperaquine concentrations on Day 7 exceeding 30 ng/mL have been shown to be predictive of therapeutic success in the treatment and prevention of uncomplicated *P. falciparum* malaria in children and adults [29, 30]. The predictive performance of the model at Day 7 was assessed by calculating the predictive error (PE) as the difference between the predicted concentration (C_pred_) and the observed concentration (C_obs_) at Day 7, and the relative difference (RD), as follows:

(4) $$\mathrm{PE}=C_{\text{pred}}-C_{\mathrm{obs}}$$

(5) $$\mathrm{RD}=\left(\frac{C_{\text{pred}}-C_{\mathrm{obs}}}{C_{\mathrm{obs}}}\right)\times 100\%$$

where C_pred_ is the predicted concentration at Day 7 for the 100 profiles simulated from the individualized profile, and C_obs_ is the observed concentration at Day 7 for the patient.

## 3 Results

**FIGURE 2:**
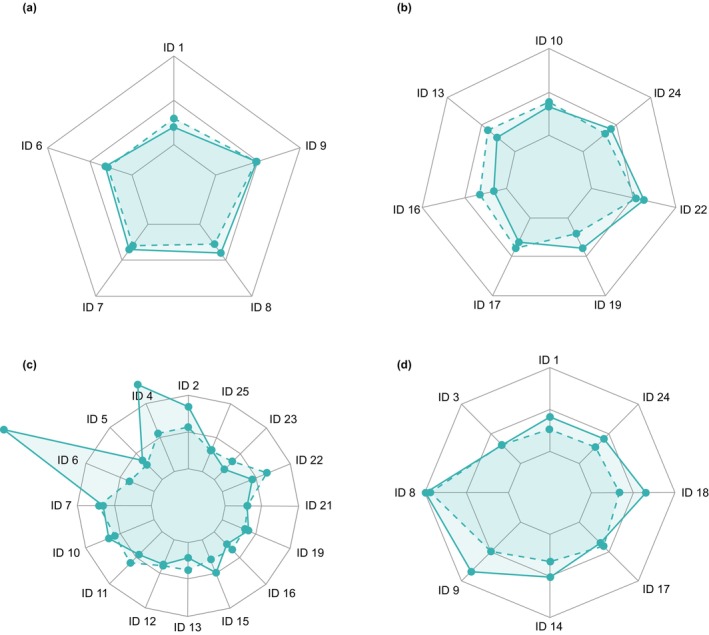
Individual piperaquine normalized AUC ratios ($R_{\mathrm{AUC}/\text{Dose}}$) for the first dose (blue solid line) and last dose (blue dotted line) in (a) Sudanese pregnant women, second trimester; (b) Sudanese pregnant women, third trimester; (c) Thai pregnant women, second trimester; (d) Thai pregnant women, third trimester. Internal gray line: $R_{\mathrm{AUC}}=0$, middle gray line: $\;R_{\mathrm{AUC}}=1$, and external gray line: $\;R_{\mathrm{AUC}}=2$. ID, identification number for the women included in the clinical trials. ID = patient identification number.

When examining the characteristics of the simulated profiles vs. the observed data, mean body weight, which was generated stochastically within the ‘Sim‐Pregnant’ population, was higher in the virtual population (75 kg, range 69–81 kg) compared to the observed data for the Sudanese population (59.6 kg, range 50–72 kg) and Thai population (49.2 kg, range 36–58 kg). As the piperaquine dose entered into the PBPK model was based on body weight (mg/kg), the total piperaquine dose administered to the individualized population was higher than the doses administered to the observed patient population, and consequently, exposures were higher (Figure S1). After dose normalization to observed body weight, *R*_AUC_ values were ≤ 2 for all 24 Sudanese women in both the second and third trimester (Figure 2A,B). For the Thai population, 14/16 in their second trimester and 8/8 in their third trimester had an *R*_AUC_ value ≤ 2 (Figure 2C,D).

**FIGURE 3:**
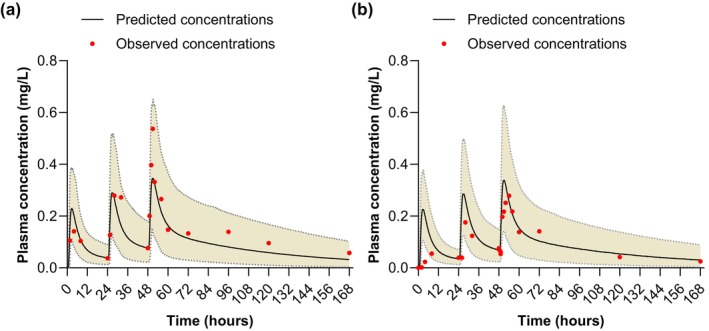
Mean plasma concentration–time plots for piperaquine in plasma in representative individualized profiles compared to observed data for (a) a Sudanese pregnant woman (second trimester, patient ID7), and (b) a Thai pregnant woman (second trimester, patient ID6). The shaded area is 95% prediction interval.

Representative predicted concentration–time profiles and observed data are shown in Figure 3. The performance of simulations was assessed according to MFE for Day 1 and Day 3 exposure to piperaquine (Table 2). The values for C_max_, AUC, and clearance (CL/F) were within the prespecified values for acceptance (> 0.5 to < 2.0), confirming that there was good agreement between the predicted and observed values up to Day 3.

**TABLE 2:** Mean fold error for piperaquine exposure values.

Exposure values	Pregnant women from Sudan			Pregnant women from Thailand		
	All (*n* = 12)	Second trimester (*n* = 5)	Third trimester (*n* = 7)	All (*n* = 24)	Second trimester (*n* = 16)	Third trimester (*n* = 8)
First dose						
AUC_0‐24_	0.63	0.67	0.60	0.82	0.77	0.98
C_max_	0.66	0.66	0.65	1.04	0.95	1.28
Last dose						
AUC_48‐72h_	0.65	0.62	0.69	0.78	0.80	0.72
C_max_	0.51	0.44	0.58	0.68	0.72	0.60

Abbreviations: AUC, area under the concentration–time curve; C_max_, maximum concentration.

**FIGURE 4:**
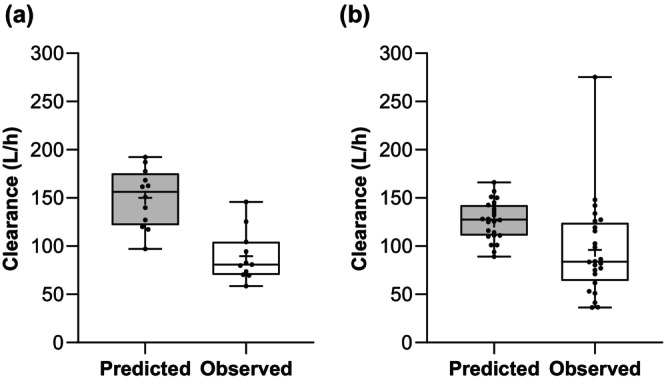
Predicted vs. observed piperaquine clearance up to Day 7 in (a) Sudanese pregnant women, and (b) Thai pregnant women. The central line is the median, the box represents the interquartile range, the cross is the mean, and the whiskers are the lowest and highest values.

**FIGURE 5:**
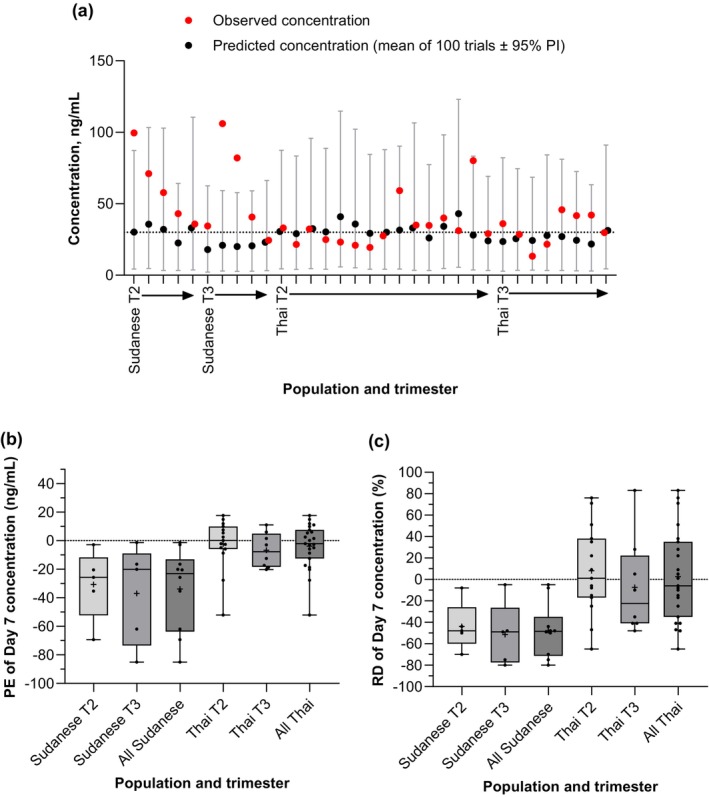
Piperaquine concentrations on Day 7 for (a) individual predicted vs. observed plasma concentrations and predictive performance assessed by (b) prediction error (PE), and (c) relative difference (RD). The central line is the median, the box represents the interquartile range, the cross is the mean, and the whiskers are the lowest and highest values. All the predicted concentrations were normalized by the actual mg/kg dose administered to the patient. T2 = second trimester; T3 = third trimester.

Considering Day 7 data, the model tended to overestimate CL/F, especially in the Sudanese population; predicted mean CL/F was 150.2 (95% CI 131.1, 169.3) L/h compared to the observed mean CL/F of 89.6 (95% CI 71.9, 107.2) L/h (Figure 4). This overestimation of CL/F in the Sudanese population was carried forward to the estimations of plasma concentrations on Day 7 (Figure 5). The predicted values were across a narrow range (18–36 ng/mL), whereas the inter‐patient variability in the observed data was greater (24–106 ng/mL). Observed values exceeded the 95% CIs of the predicted values for three of the ten patients; these were also the patients with the highest observed Day 7 concentrations. Even considering the small sample size (*n* = 10), there was a pattern of underprediction of Day 7 concentrations for the Sudanese population, with a mean percent error of −47.7% (SD 23.9%).

For the Thai population, at Day 7, there was closer agreement in CL/F; predicted mean CL/F was 126.6 (95% CI 118, 135) L/h, and observed was 96.3 (95% CI 74.9, 117.6) L/h, and this was reflected in the similar predicted and observed Day 7 exposures in the Thai population (Figure 5).

## 4 Discussion

Clinical trials in pregnant women are rarely conducted. However, malaria can have serious and even fatal consequences to pregnant women, the fetus, and neonates [31, 32, 33]. The impact of pregnancy and malaria on drug absorption, distribution, metabolism, and excretion must be understood to assess the risk of adverse events or reduced efficacy for the mother due to increased or decreased exposure to the drug, respectively, and to identify any potential impacts on the fetus and child development [34]. Thus, as part of the Malaria in Mothers and Babies (MiMBa) initiative, MMV Medicines for Malaria Venture is investigating approaches for more expeditious drug development while ensuring patient safety [6]. PBPK modeling is supporting this initiative, as it can be applied to predict drug PK in pregnant women and thereby guide potential dose adjustments and drug development strategies [35].

This exploratory methodological study used an individualized PBPK modeling approach to predict exposures of the antimalarial drug piperaquine in pregnant women in their second and third trimesters and compare them with clinical data in the same populations. The study was not designed to inform piperaquine dose adjustment. Piperaquine was chosen because a rich PK data source was available in the target population, and a PBPK model was verified in Simcyp for healthy adults. Also, piperaquine is primarily metabolized via cytochrome P450 3A4 (CYP3A4) [36] and represents a good case study for other CYP3A4 substrates [37]. The study aimed to create an individualized virtual profile and measure the performance of the simplest possible approach to assess whether the procedure would be useful and accessible in the clinic.

The simulated exposures until Day 3 were generally in agreement with the clinical observations (within two‐fold) for both the Sudanese and Thai populations, showing good model specification. However, this was dependent upon *post hoc* dose normalization of predicted AUC because the Simcyp pregnancy population generated higher body weights than those observed in either clinical population. Although age and piperaquine dose could be specified in the virtual trial design, there was no straightforward method to individualize body weight. Even though we re‐expressed exposures on a consistent per‐kilogram basis aligned with individual patient characteristics, we recognize that this is not ideal, and individualization of body weight would be preferable. However, it does illustrate that care is needed in applying simulated population data, which is based on Caucasian women, to women living in malaria‐endemic countries.

Clinical studies show that patients with piperaquine plasma levels below 30 ng/mL on Day 7 were more likely to have a recurrence of malaria caused by either *P. falciparum* or *P. vivax* [29]. Also, for the intermittent preventive treatment of malaria with DHA‐PPQ, having a Day 7 piperaquine plasma concentration < 30 ng/mL was significantly associated with a higher risk of parasitemia during pregnancy (*p* = 0.004) [38]. In the clinical setting, measurement of the plasma concentration of piperaquine on Day 7 is simpler than obtaining frequent plasma concentration samples to calculate AUC. Plasma concentration at Day 7 can therefore be used as a PK marker of efficacy. In the individualized PBPK model, Day 7 concentrations were reasonably specified for the Thai population. However, the model tended to overestimate predicted CL/F at Day 7 in the Sudanese population. The overestimation of CL/F at Day 7 resulted in an underestimation of the predicted plasma concentrations at Day 7 in the Sudanese population. Note that the observed median [range] piperaquine half‐life was very similar in Sudanese pregnant women (17.9 [11.1–29.0] days) and Thai pregnant women (17.8 [8.88–24.9] days) [25, 26].

The explanation for the increased predicted CL/F is unknown, but similar findings were noted previously in the PBPK model for piperaquine in non‐pregnant women [39]. In that case, the model was built using data specifically for the *Eurartesim* DHA‐PPQ formulation to assess infant exposures in breastmilk. However, predictions underestimated the terminal half‐life when compared against a range of clinical studies [39], and model refinement was needed to ensure adequate recovery of the drug concentrations in plasma before predicting milk concentrations [39]. There are currently no explanations for this discrepancy in clinical PK data, and the decision was made to use the original *Eurartesim* piperaquine (fasted) model in the current study. Piperaquine has a very large volume of distribution, multi‐compartment kinetics, and a long terminal half‐life. Although sensitivity analysis around these parameters could be considered, given the limited clinical data, performing exploratory sensitivity analyses without external validation could risk overfitting the model to the current dataset.

Other possible explanations for the overestimation of Day 7 CL/F derive from the simulated population being healthy pregnant women, whereas the observed population had malaria. Acute malaria is associated with reduced CYP3A4 activity due to inflammation, anemia, altered hematocrit, and increased levels of alpha‐1‐acid glycoprotein [40, 41]. The reduced CYP3A4 activity would be expected to reduce CL/F in the clinical population, and so is consistent with the model findings. A decrease in piperaquine oral clearance of ≥ 35.9% has been observed in mice in a malaria infection model [42]. However, malaria resolution would restore CYP activity, increasing clearance, contrary to the model findings. Quantitative incorporation of these effects would require time‐based CYP activity data in pregnant malaria patients, which are currently lacking. A preliminary virtual population model of (non‐pregnant) patients with uncomplicated malaria has been developed by Simcyp in collaboration with MMV Medicines for Malaria Venture and is available for Simcyp users for exploratory evaluation (https://pbpkrepository.certara.co.uk/). An area for further study would be the development of a virtual population of pregnant women with malaria with the aim of improving predictive accuracy. This would have broad application across the development and evaluation of antimalarials for use during pregnancy. Understanding the strengths and limitations of the pregnancy and malaria models independently is a key step towards a combined model.

It is not clear why a disease effect would have a greater impact on CL/F in Sudanese vs. Thai women. This suggests that there may be population‐based differences, such as genetic or environmental influences on drug metabolism, CYP3A4 activity, body composition, nutritional status, and disease‐related factors such as parasitemia level, which can vary based on the transmission setting.

Another consideration is that, although the simulations accounted for dosing in the fasted state, this information was missing for the study conducted in Thailand, and so could not be explored in the model. Clinical studies indicate higher variability of piperaquine PK when given in the fasted state, and a high‐fat meal increases exposure [43]. Although the study in Sudanese patients dosed piperaquine in the fasted state, differences in food intake after dosing could have impacted piperaquine pharmacokinetics, though this should have been evident in the Day 3 exposures. In the Sudanese population, the model did not reflect the high observed inter‐patient variability. Ongoing work is seeking to further elucidate the underlying mechanisms driving piperaquine PK variability and non‐linearity, which could then be incorporated into the model. In this case, piperaquine was used as a case study example of an individualized PBPK approach, and our findings should not be used to inform considerations of dose adjustment in pregnancy.

The key strength of using an individualized profiles approach is that it allowed PBPK model simulations to be compared for each patient. To our knowledge, this approach has not been explored previously for the investigation of antimalarial drugs in any population. The ‘virtual twin’ approach has been previously used to predict olanzapine exposure [19], and to determine gene–environmental effects of patients treated with clozapine [17, 18], and more recently with apixaban or rivaroxaban [21]. In an evaluation using a caffeine PBPK model, matching individual patient characteristics considerably improved the simulation results vs. a standard population‐based model [14]. So far, only one published study has included pregnant women, with the individualized maternal model performing well, demonstrating that CYP2D6 activity largely affected risperidone and paliperidone PK during pregnancy [16]. Overall, individualized PBPK modeling appears to be a useful approach in populations where clinical data may be difficult to obtain, such as pregnant women. An extension of this approach would be to investigate drug–drug interactions in pregnant women. For example, pregnancy and the administration of the HIV drug efavirenz both independently reduce concentrations of the antimalarial drug lumefantrine, and understanding the dosing implications of this combined effect would be beneficial [44, 45].

The major limitation was that body weight was not individualized in the virtual profiles and was overestimated using the Simcyp library pregnancy population. Noting that piperaquine PK may not be dose‐linear at higher concentrations [46], given the narrow range of therapeutic piperaquine doses administered in the clinical trials, a linear adjustment was acceptable. Although there is scope to further refine the model approach by integrating additional individualized parameters, including body weight, this can be methodologically complex. For example, body weight was not individualized because this requires specification of a body weight to height function in Simcyp. The feasibility of the individualized approach could be explored for other platforms, e.g., Open Systems Pharmacology has a PBPK pregnancy model [47], though the piperaquine model in healthy non‐pregnant adults would require transfer and reverification.

The goal of PBPK simulations for pregnant populations is to adjust, when needed, the doses administered to pregnant women during initial clinical trials, and then possibly for clinical use. This is key to avoiding reduced efficacy when the exposures are predicted to be lower, or safety risk when the exposures are predicted to be greater in pregnant women. In the clinical study comparing piperaquine PK profiles in pregnant vs. non‐pregnant women, there was a trend of lower exposures in pregnant women [25]. In cases where the difference in exposure might be more significant, being able to anticipate this would allow dose adjustment prior to administration. However, dose adjustment should consider both total plasma exposures and free concentrations, which represent the drug available for efficacy. For example, piperaquine is highly protein‐bound [48], presumably to alpha‐1 acid glycoprotein [27], and plasma protein binding varies during pregnancy [49]. Therefore, differences in total drug exposures may not be directly linked to therapeutic responses, which are driven by free concentrations. The PBPK model incorporates changes in pregnancy binding proteins, and predictions of both total and free drug PK should be considered if dose adjustment is envisaged. However, most clinical studies do not report free drug concentrations, limiting the opportunities for model verification against observed data.

In conclusion, understanding the PK of antimalarial drugs in pregnant women is essential, given the severe risks malaria poses to this population, the fetus, and newborns. This study employed an individualized profile PBPK modeling approach to predict piperaquine exposures in pregnant women. Over the three‐day dosing schedule, the results closely aligned with clinical observations. While the methodology showed promise, the overestimation of CL/F at Day 7, especially in the Sudanese population, underscores the need for more tailored models that reflect demographic, genetic, environmental, and physiological variations. The current findings highlight both the potential advantages and the limitations of individualized PBPK modeling in pregnancy, emphasizing the need for additional clinical PK data, particularly over extended sampling periods. This work contributes to the development of PBPK models that predict drug PK in pregnant women to support their early inclusion in drug development and clinical trials to ensure patient safety and effective treatment outcomes in this population.

## Author Contributions

S.K. designed and performed the research. S.K. and N.A. analyzed the data. S.K., N.A. M.E.G. and J.T. wrote the manuscript.

## Funding

The authors have nothing to report.

## Conflicts of Interest

S.K. and J.T. declared no competing interests for this work. N.A. and M.E.G. are employees of MMV Medicines for Malaria Venture.

## Supporting information


**Table S1:** Inputs for the piperaquine phosphate compound file in Simcyp [1].

**Figure S1:** Piperaquine AUC ratios for the first dose and last dose unadjusted for body weight. (a) Sudanese woman, second trimester; (b) Sudanese woman, third trimester; (c) Thai woman, second trimester; (d) Thai woman, third trimester.

## Data Availability

Data are available on reasonable request and with completion of a signed data access agreement from (https://www.mmv.org/about‐us/contact‐us) referencing this publication. Data will be available for at least five years from publication of this study.
