# Supplementary material for: Physiologically‐Based Pharmacokinetic Modeling to Investigate Piperaquine Exposure in Pregnant Women Using an Individualized Profile Approach
Source: Clin Transl Sci. 2026 May 12;19(5):e70589. doi: 10.1111/cts.70589 (PMC13163144; doi:10.1111/cts.70589)
Supplement: Supplementary file 1 — Table S1: Inputs for the piperaquine phosphate compound file in Simcyp [1]. [file CTS-19-e70589-s002.docx]

# TABLE S1. Inputs for the piperaquine phosphate compound file in Simcyp™ [1].

| Parameter | Value | Source |
| --- | --- | --- |
| Physicochemical |  |  |
| Molecular weight (g/mol) | 535.52 | PubChem |
| Log P_o:w_ | 5.27 (Diprotic base) | Calculated [2] |
| pKa1 | 8.8 | Calculated [2] |
| pKa2 | 7.4 | Calculated [2] |
| Blood binding |  |  |
| Blood:Plasma | 0.90 | Unpublished data |
| fu_p_ | 0.006 | Unpublished data |
| Absorption (first order model) |  |  |
| fa (fasted) | 0.4 | European Medicines Agency [3] |
| ka (h-1) | 0.6 | Optimized to recover clinically observed data |
| tlag (h) | 1.0 |  |
| Q_gut_ (L/h) | 18.6 | Predicted [4] |
| Caco-2 (P_app_ a⭢b) 10^-6^ cm/s | 233 | Calculated [2] |
| fu_gut_ | 0.006 | Set to equal fu |
| Distribution (full PBPK) |  |  |
| Vss (L.kg^-1^) | 47.4 | Predicted [5] |
| Kp scalar | 3 | Optimized to recover concentration–time profile in the rat [6] |
| Metabolism |  |  |
| CYP3A4, HLM, CL_int_, μmol/min/mg | 25.28 | HLM CL_int_ 31.6 µL/min/mg with 80% assigned to CYP3A4, thus fmCYP3A4 = 80%; based on unpublished chemical inhibition data |
| CYP2C9, HLM, CL_int_, nmol/min/mg | 3.16 | HLM CL_int_ 31.6 µL/min/mg with 20% split equally between CYP2C9 and CYP2C19, thus fmCYP2C9 = 10% and fmCYP2C19 = 10%, European Medicines Agency [3] |
| CYP2C19, HLM, CL_int_, nmol/min/mg | 3.16 |  |
| Fu_mic_ (for all CL_int_) | 0.013 | Calculated [2] |
| CYP3A4 Ki (µM) | 3.0 | Calculated [2] |
| CYP3A4 k_inact_ (h^-1^) | 1.25 | Derived from unpublished IC_50_ shift data |
| CYP3A4 K_l_ (µM) | 0.12 | Derived from unpublished IC_50_ shift data |
| Elimination |  |  |
| CL renal (L/h) | 0 | Assumed |

Log P_o:w_, octanol/water partition coefficient; pKa, the pH at which the ionized and unionized forms exist in equal concentrations for a particular pH; fu_p_, fraction unbound in plasma; fa, fraction of the orally administered dose absorbed into the bloodstream in the fasted state; ka, first order absorption rate constant; tlag, lag time before absorption starts; Q_gut_, blood flow rate to the gut; Caco-2 (P_app_ a⭢b), apparent permeability coefficient across Caco-2 cell monolayers; fu_gut_, fraction unbound in the gut; V_ss_, clearance independent volume of distribution; Kp, predicted tissue-to-plasma partition coefficients; CYP, cytochrome P450; HLM, human liver microsome; CL_int_, intrinsic clearance; Fu_mic_, fraction unbound in microsomes ; K_i_, inhibition constant; k_inact_, maximum rate of enzyme inactivation for mechanism-based inhibitors; K_l_, concentration at which half-maximal inactivation occurs; CL renal, renal clearance.

**Source references**

1. N. Abla, E. Howgate, K. Rowland-Yeo, et al, "Development and application of a PBPK modeling strategy to support antimalarial drug development," *CPT Pharmacometrics and Systems Pharmacology* 12 (2023): 1335-1346.

2. S. A. Charman, A. Andreu, H. Barker, et al, "An in vitro toolbox to accelerate anti-malarial drug discovery and development," *Malaria Journal* 19 (2020): 1.

3. European Medicines Agency, "Eurartesim dihydroartemisinin / piperaquine phosphate assessment report," (2011), <https://www.ema.europa.eu/en/documents/assessment-report/eurartesim-epar-public-assessment-report_en.pdf>. A. Accessed: 7 April, 2025.

4. J. Yang, M. Jamei, K. R. Yeo, et al, "Prediction of intestinal first-pass drug metabolism," *Current Drug Metabolism* 8 (2007): 676-684.

5. T. Rodgers, M. Rowland, "Physiologically based pharmacokinetic modelling 2: predicting the tissue distribution of acids, very weak bases, neutrals and zwitterions," *Journal of Pharmaceutical Sciences* 95 (2006): 1238-1257.

6. J. Tarning, N. Lindegardh, S. Sandberg, et al, "Pharmacokinetics and metabolism of the antimalarial piperaquine after intravenous and oral single doses to the rat," *Journal of Pharmaceutical Sciences* 97 (2008): 3400-3410.
