# Supplementary material for: Physiologically‐Based Pharmacokinetic Modeling to Investigate Piperaquine Exposure in Pregnant Women Using an Individualized Profile Approach
Source: Clin Transl Sci. 2026 May 12;19(5):e70589. doi: 10.1111/cts.70589 (PMC13163144; doi:10.1111/cts.70589)

# Figure S1. Piperaquine AUC ratios for the first dose and last dose unadjusted for body weight.

(a) Sudanese woman, second trimester; (b) Sudanese woman, third trimester; (c) Thai woman, second trimester; (d) Thai woman, third trimester.

Blue solid line: AUC ratio for first dose; blue dotted line: AUC ratio for last dose; internal grey line: RAUC=0; middle grey line RAUC=1; external grey line RAUC=2. ID, identification number for the women included in the clinical trials.


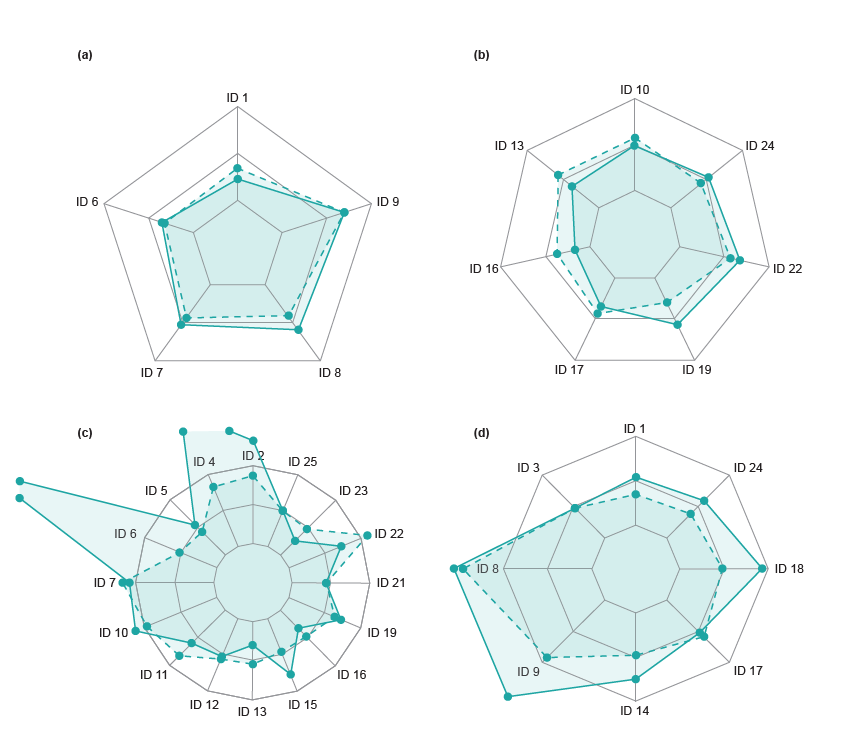

Supplement: Supplementary file 2 — Figure S1: Piperaquine AUC ratios for the first dose and last dose unadjusted for body weight. (a) Sudanese woman, second trimester; (b) Sudanese woman, third trimester; (c) Thai woman, second trimester; (d) Thai woman, third trimester. [file CTS-19-e70589-s001.docx]
